# Skin tone and clinical dataset from a prospective trial on acute care patients

**DOI:** 10.1038/s41597-025-06457-9

**Published:** 2026-01-28

**Authors:** Sicheng Hao, Joao Matos, Katelyn Dempsey, Mahmoud Alwakeel, Jared Houghtaling, Chuan Hong, Judy Gichoya, Warren Kibbe, Michael Pencina, Christopher E. Cox, An-Kwok Ian Wong

**Affiliations:** 1 Duke University School of Medicine, Department of Medicine, Division of Pulmonary, Allergy, and Critical Care Medicine, Durham, NC USA; 2 Duke University School of Medicine, Department of Biostatistics and Bioinformatics, Division of Translational Biomedical Informatics, Durham, NC USA; 3 The Institute for Clinical Research and Health Policy Studies at Tufts Medical Center, Medford, USA; 4 Emory University, Department of Radiology and Imaging Sciences, Atlanta, GA USA; 5 Duke University School of Medicine, Duke AI Health, Durham, NC USA; 6 Durham VA Medical Center, Durham, NC USA

**Keywords:** Health care, Medical research

## Abstract

Although hypothesized to be the root cause of the pulse oximetry disparities, skin tone and its use for improving medical therapies have yet to be extensively studied. Studies that previously used self-reported race as a proxy variable for skin tone cannot account for skin tone variabilities within race groups. This study aimed to create a unique baseline dataset that included skin tone and electronic health record (EHR) data to better evaluate health disparities associated with pulse oximetry. We collected skin tone data at 16 different body locations using multiple devices, including administered visual scales, colorimetric, spectrophotometric, and photography via mobile phone cameras. All patients’ data were converted into a common data model and de-identified before publication in PhysioNet. We assessed 167 features per skin location on 128 patients linked with their EHR data, such as laboratory data, vital sign recordings, and demographic information. We also include 2,438 images from mobile phones to assist in developing artificial intelligence tools to combat health disparities.

## Background & Summary

Pulse oximetry is a fundamental non-invasive tool used in healthcare settings worldwide that enables monitoring of oxygen saturation levels and heart rate through light-emitting diodes at specific wavelengths (660 nm and 940 nm)^1^. By measuring differences in light absorption by oxyhemoglobin and deoxyhemoglobin, this technique, estimates the peripheral oxygen saturation (SpO_2_) as a surrogate for arterial oxygen saturation (SaO_2_) which in turn provides critical data for patient triage, monitoring, and intervention^2^. However, this fundamental dependence on optical technology also makes it susceptible to various factors that can skew readings, such as patient movement, ambient light interference, and, most critically, variations in skin pigmentation^3–9^. As such, pulse oximetry’s reliability has become increasingly questioned, especially in critical care settings where accurate measurements are vital^10^.

Recent studies have highlighted significant racial and ethnic discrepancies in SpO_2_ readings, which falsely minimize the actual severity of hypoxemia in individuals with darker skin tones^3,11^. These inaccuracies in pulse oximetry can lead to mismanagement of critically ill patients, resulting in severe consequences, including organ dysfunction and death^12^. Furthermore, these discrepancies often result in delayed or inadequate interventions, exacerbating health inequities and complicating clinical decision-making^3–9^. Studies also suggest that conventional calibration methods for pulse oximeters may not account for physiological variations across different skin tones, underscoring the need for tailored approaches^13–15^. The underlying issue has been traced to differences in skin tone, which affect the oximeter’s light absorption readings. Yet, the integration of skin tone as a medical variable has been limited, leading to a substantial knowledge gap in how pulse oximetry should be adjusted or interpreted based on this factor^7,16^.

To bridge this gap, our research, encapsulated in the ENCoDE (mEasuring skiN Color to correct pulse Oximetry DisparitiEs) study, aims to systematically collect and analyze skin tone data at different body locations from patients requiring acute care. This study is designed to develop a robust framework for incorporating skin tone into clinical assessments. We measured over 167 skin tone features, including both subjective and objective measurements (details in Table 1), on different skin locations and put them side by side, creating opportunities for a thorough investigation of the advantages and disadvantages of various methods for measuring patients’ skin tone. By linking these findings with clinical data in electronic health records (EHR), potential opportunities arise for researchers to investigate healthcare disparities associated with skin tone, thereby enhancing patient care and outcomes. Although collected from a single medical center due to limited resources, this dataset is mapped to the OMOP Common Data Model, allowing ease of reusability and harmonization for future multi-center datasets. Together with smartphone images collected for ten non-biometric body locations, this dataset could potentially assist the collaboration between medical researchers and the medical AI communities to identify and combat skin tone-associated disparities, as well as provide more exploration that can guide regulatory bodies in evaluating pulse oximetry devices.

**Table 1:** Structured skin tone features.

Device (type)	Measurement space	Measurements	Number of features
Administered Visual Scales (card)	Categorical skin tone scales	Fitzpatrick scale	1
		Von Luschan’s Chromatic Scale	1
		Monk SkinTone Scale	1
Delfin SkinColorCatch (colorimeter)	CIE	L*a*b*	3
	Color Index	Melanin Index; Erythema Index, Individual Typology Angle (ITA)	3
Konica Minolta CM700d (Spectrophotometer)	CIE	XYZ, L*a*b*, L*C*h	8
	Hunter L*a*b*	L*, a*, b*	3
	Munsell color system	Hue, value, chroma	3
	Spectrum	400 nm - 700 nm, every 10 nm	31
Variable Inc. Spectro 1 Pro (Spectrophotometer)	RGB, under different standard illuminants*	i.e., A-2deg-Red^1^	
	24		
	CIE L*a*b, under different standard illuminants*	i.e., F2-10deg-L*	24
	Hex, under different standard illuminants*	Color codes (i.e., #907356)	8
	Spectrum	400 nm–700 nm, every 10 nm	31
Processed from mobile devices (iPhone SE 2020, Google Pixel 4a)	CIE	Average and standard deviation of the intensity for each channel in red, green, blue, and CIE L*C*H	24

This table describes all the features of skin tone measurement we collected in our dataset. Measurement devices and methods include administered visual scales (Fitzpatrick Skin Type, Monk Skin Tone, and Von Luschan); reflectance colorimetry (Delfin SkinColorCatch, Kuopio, Finland); and reflectance spectrophotometry (Variable Spectro 1 Pro Bridge Set, Variable, Inc TN, USA. and Konica Minolta CM-700D Spectrophotometer, Tokyo, Japan).

In “A-2deg-Red”, “A” is the white point of standard illuminants, “2 deg” is the field of view, which is short for 2 degrees, and “Red” is the color component of the RGB representation.

## Methods

### IRB

The Institutional Review Board at Duke University Medical Center reviewed patient information collection and creation of the research resource, which granted a waiver of informed consent and approved the data-sharing initiative under Pro00110842 on 18 May 2022, titled “ENCODE (mEasuring skiN Color to correct pulse Oximetry DisparitiEs).”

### Cohort acquisition

The ENCoDE project enrolled patients admitted to inpatient care at Duke University Hospital (Durham, NC, USA) with hospital encounters. The requirement for inclusion was synchronized ABG-pulse oximetry measurements, defined as at least one pulse oximetry value recorded within 5 minutes prior to an ABG value captured in EHR, and referred to as SaO_2_-SpO_2_ pairs following the standard of previous observation study^3^. All data, including patient consent, measurements, and EHR data, were stored in REDCap electronic data capture tools hosted at Duke University^17,18^. For patients unable to consent, a Legally Authorized Representative (LAR) would consent on their behalf, and the patient re-consented after they regained the ability to consent. Exclusion criteria included unremovable fingernail polish, admission for vascular complications (e.g., grafting or stenting), any limb amputations, and causes of skin discoloration such as vitiligo, jaundice, and wounds/bruising. These criteria were established to ensure data quality by avoiding cases of arterial insufficiency or other conditions that could affect skin tone measurement across all patient locations.

### Data collection

#### Skin tone and skin temperature data collection

In all patients, four modalities of skin assessments were conducted: infrared temperature (using the HoMedics HTD8813C [clinical range, 34–42.9 °C] and IDEAL Model #61–847 [general range, −32–500 °C]), administered visual scales (Fitzpatrick Skin Type, Monk Skin Tone, and Von Luschan), colorimetric (Delfin SkinColorCatch), spectrophotometric (Konica-Minolta CM-700d, Variable Spectro 1 Pro), and photography via mobile phone cameras (Google Pixel 4a, iPhone SE 2020). Visual skin scales were printed for reference on 4“x6” photo paper.

Measurements were taken using all four skin assessment modalities at sixteen different locations: eight on the left and right upper extremities (dorsal and ventral finger pad, dorsal and ventral palm), three on the head (forehead, inner and outer surface of an earlobe), one on the sternum, and four on the left and right lower extremities (dorsal and ventral toe). Measurements were collected from patients lying down or in a seated position. A black card was placed on the opposite side for earlobes (and fingers, if needed) to reduce the impact of reflection. For this study, measurements were collected from patients admitted to an intensive care unit (ICU) and regular floor units.

The study utilized two trained personnel to collect measurements to improve the timeliness of data collection and create an efficient workflow in a dynamic, multidisciplinary, and fast-paced environment. The location of the pulse oximeter was reported as directly observed by the clinical research coordinator at the time of data collection or surveyed by clinical staff regarding location at the time of the ABG.

#### EHR data

Patients’ hospital encounter information, demographic details, laboratory measurements (such as arterial blood gas panel, complete blood count panel, and comprehensive metabolic panel), and flowsheets (containing measurements of standard vital signs and information about oxygen delivery) were extracted from Duke University Hospital’s EHR system (EPIC Clarity). All data, including image records, were linked to the patient’s encounter using unique hospital account identifiers.

### Data processing

#### Data linking

To link skin tone data with patients’ EHR data, we extracted all structured data from EPIC Clarity and REDcap into Duke’s Protected Analytics Computing Environment (PACE) for processing. Tables from the two systems are linked via the hospital encounter number and the patient’s medical record number (MRN).

#### Image feature processing

Every image captured by mobile devices underwent a filtering process to isolate the brightest section, achieved by selecting the largest contour above the median brightness in greyscale. Subsequently, a mask was generated to eliminate extraneous values. An example processed image, with full consent from the measurement subject, is provided below (Fig. 2). From these masked images, the average and standard deviation on each RGB (Red, Green, and Blue) and LCH (Lightness, Chroma, and Hue) channels were extracted as features linked to the patient’s skin tone. All the pixel intensities from camera images were retained unaltered to ensure potential data users could access the untransformed data.

#### De-identification

We de-identified our dataset according to provisions of the Health Insurance Portability and Accountability Act (HIPAA) Safe Harbor. All date and time information was shifted randomly into the future while preserving the difference within one subject. Patient encounter numbers and MRNs were randomly remapped to visit_occurrence_id and patient_id. All measurement values were first evaluated as strings and then whitelisted before deidentification. Image data from potential biometric locations (ventral side of both left and right fingers, palms, and toes) are excluded. However, processed features (e.g., mean or standard deviation RGB values) from images that are not considered biometric information are included.

#### OMOP conversion

**Fig. 1:**
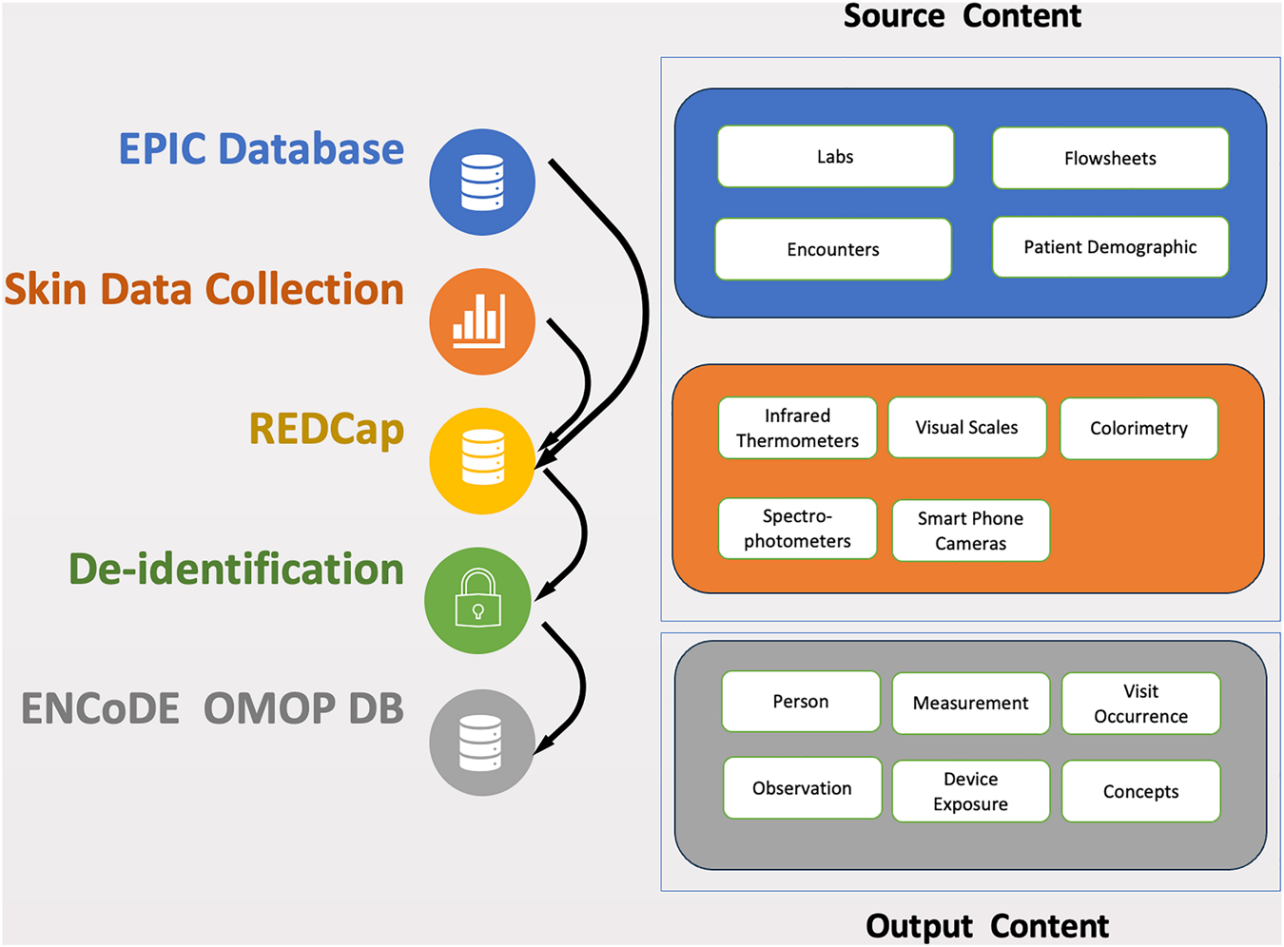
Data flow diagram and content. The left side of the figure represents how data flows in the collection process. Firstly, EHR data are pulled from EPIC databases into REDCap, and patient skin data is collected at the bedside and stored in REDCap. Then, the data are de-identified before leaving Duke’s compute enclave PACE via an honest broker request. Lately, data has been transformed into an OMOP format. The right side of the figures provides a high-level view of the data content. Source content contains patient’s EHR tables and data from five different types of devices or collection methods. Output content contains tables and images in OMOP format.

The Observation Medical Outcomes Partnership (OMOP) Common Data Model (CDM) is a standardized framework designed to enable systematic analysis of disparate observational healthcare databases, facilitating large-scale data integration and research. We converted our structured tables into OMOP format following OMOP CDM version 5.4^19^. We manually mapped semantic concepts to standard representations in the OMOP vocabularies; these mappings were then validated by two clinical experts. For source concepts without an existing standard representation, such as reflectance measurements of skin tone at a particular location with a specific device, we created custom standard concepts that we will eventually contribute back to the OMOP vocabulary team for future uptake into the community-curated vocabularies. In total, we created 2,704 concepts to represent various imaging-related elements specific to this study. With regard to the data model itself, this dataset includes rows in the following tables: PERSON, VISIT_OCCURRENCE, MEASUREMENT, OBSERVATION, DEVICE_EXPOSURE, PROCEDURE_OCCURRENCE, OBSERVATION_PERIOD, and CDM_SOURCE. A comprehensive summarization of the data flow can be found in Fig. 1.

**Fig. 2:**
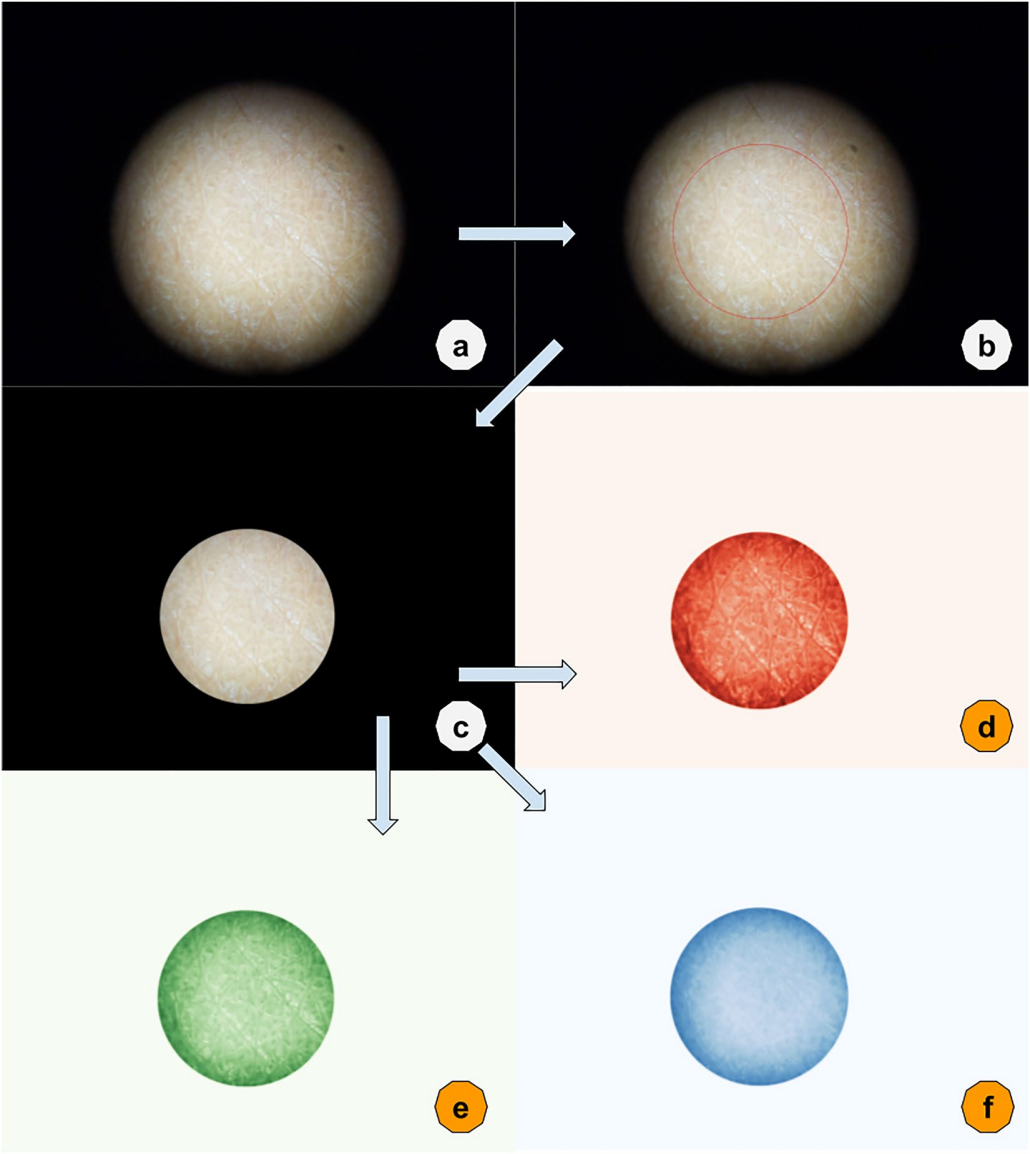
Image processing. This figure demonstrated how raw images were processed. (**a**) is the raw image taken with smartphone cameras. (**b**) The circle was calculated based on brightness representing the center of the image. (**c**) is the image output to the dataset, and information inside the circle from (2) is kept. (**d–f**) are representations of how to derive image figures such as average red, green, and blue from the output image (**c**).

## Data Records

ENCoDE is available on PhysioNet as a credentialed database^20^. (https://physionet.org/content/encode-skin-color/1.0.0/).

### Cohort and EHR data

**Fig. 3:**
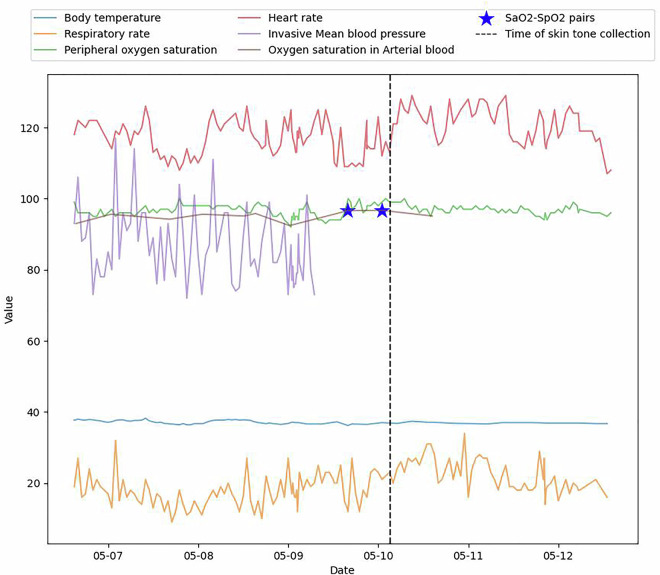
Sample data for a single patient. This is a timeline plot of a single patient’s data selected at random. The gold star represents the SaO_2_ - SpO_2_ pair we collected before skin data collection. The dashed black line represents the beginning of skin data collection. EHR data are available before and after skin collection.

From January 2023 to June 2023, a total of 1,119 admitted inpatients with qualifying SaO_2_-SpO_2_ pairs were screened at Duke University Hospital. Out of those patients, 302 met our inclusion criteria and were approached, of whom 134 consented to this study. After six exclusions due to withdrawal or missing skin tone data, 128 patients were included in the final cohort. (39.8% female, 43% Black). EHR data associated with the patient cohort are released using the standard OMOP format. Figure 3 visualizes a few selected clinical features for one hospitalized patient.

### Skin tone features

A total of 167 skin tone features from three administered visual scales (Fitzpatrick Skin Type, Von Luschan, Monk Skin Tone), a colorimetry device (Delfin Technologies, SkinColorCatch), two spectrophotometer devices (Konica Minolta CM700d; Variable Inc, Spectro 1 Pro), two thermometers, and processed features from two types of mobile phone cameras (iPhone SE, hereafter referred to as iPhone; Google Pixel 4a, hereafter referred to as Android) were collected (more details in Table 1). As a medical concept, skin tone measurements have yet to be extensively investigated, and standardized concepts for skin tone at various locations are currently absent in the OMOP vocabulary. Therefore, skin tone concepts were created for this study. All skin tone and skin temperature measurements were transformed into a long format and incorporated into the OMOP “measurement” table. These novel concepts were captured as OMOP’s “unit_concept_id” with skin tone at a specific location as “measurement_concept_id” (e.g., measurement: unit:). Additionally, skin temperature was measured at the same locations using one clinical-range [clinical-range, 34 - 42.9 °C] temperature measurement device and one general-range [general-range, −32–500 °C] temperature measurement device.

### Smartphone images

To prevent the potential leak of Protected Health Information (PHI) for biometric imaging data, we released processed smartphone image data from 10 out of 16 body locations, excluding any territory containing finger, palm, or toe prints. With images missing due to other reasons described below, the open-source data files contain 1227 Android images and 1211 iPhone images.

### Missing data

Missing data occurred occasionally in the smartphone data collection and two spectrophotometer devices due to technical issues or patient refusal. Twenty-nine patients are missing Variable Spectro 1 Pro measurements, and eight patients are missing Konica Minolta CM700d measurements. Detailed missingness rates for skin tone measurements can be found in Table 2. In the merged EHR clinical data, missingness occurred in vital signs and laboratory test values when no value was found within the set windows.

**Table 2:** Characteristics of the study cohort.

		Grouped by Race				
		Missing	Black	Other	White	Overall
**n**			57	15	56	128
**Ethnicity, n (%)**	Not Hispanic/Latino	0	57 (100.0)	12 (80.0)	52 (92.9)	121 (94.5)
	Hispanic/Latino			1 (6.7)	3 (5.4)	4 (3.1)
	Unknown			2 (13.3)	1 (1.8)	3 (2.3)
**Gender, n (%)**	Female	0	23 (40.4)	3 (20.0)	25 (44.6)	51 (39.8)
**Oximeter location, n (%)**	Finger (include missing)	0	54 (94.7)	14 (93.3)	56 (100.0)	124 (96.9)
	Forehead		1 (1.8)			1 (0.8)
	Toe		2 (3.5)	1 (6.7)		3 (2.3)
**First ICU, n (%)**	MICU	7	9 (16.4)	3 (20.0)	12 (23.5)	24 (19.8)
	SICU		30 (54.5)	9 (60.0)	29 (56.9)	68 (56.2)
	other ICU		16 (29.1)	3 (20.0)	10 (19.6)	29 (24.0)
**ICU LoS, median [Q1,Q3]**		38	4.3 [2.0,11.1]	2.3 [0.9,3.1]	5.2 [2.4,8.7]	4.1 [1.8,9.3]
**Hospital LoS, median [Q1,Q3]**		0	21.0 [9.0,39.0]	10.0 [5.5,13.5]	16.0 [7.8,33.0]	16.5 [8.0,33.2]
**# SaO**_**2**_**/SpO**_**2**_ **Pairs, median [Q1,Q3]**		0	2.0 [1.0,4.0]	1.0 [1.0,2.0]	3.0 [1.0,6.2]	2.0 [1.0,5.0]

Demographic information for all 128 patients, along with their skin tone measurements, were grouped by race. The group “Other” contains patients who self-identify as Asian (n = 5), American Indian / Alaskan natives (n = 6), More than two races (n = 2), and Unknown race (n = 2).

## Technical Validation

Skin tone data were collected and stored in REDCap^17^. Collection of all skin tone records was tracked via an audit trail system while maintaining HIPAA compliance status, and all data are exported using a version-controlled automated procedure.

All skin color data were collected in a controlled environment, to account for the unpredictability and dynamic environment of the ICU. All blinds were shut, and all light sources were turned on surrounding the patient’s bed.

ENCoDE data processing and de-identification follow the best practices of open-source databases such as MIMIC-IV^21^. The database’s build process is documented in the version-controlled environment, with continuous data validation throughout the build process. The validation process includes checks for data integrity, where patient data are linked correctly in each of the OMOP tables; data plausibility, where the information recorded is validated against a range of typical physiological values; and data privacy, where protected health information (PHI) is identified and redacted from the dataset with the help of Duke’s Honest Broker system.

**Fig. 4:**
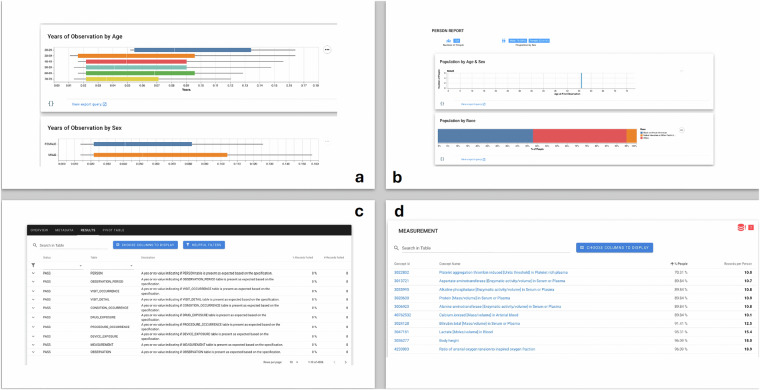
Samples of data explorations performed by ARES. In this figure, (**a,b**) are part of the output from explorations of the Observation table and Person table. (**c**) is a detailed view of a subset of all the quality checks mentioned in Table 3. (**d**) is a subset of all the items in the Measurement table ranked by lowest appearance rate among all patients. Detailed information on the whole dashboard can be found on the GitHub page: aiwonglab/ENCoDE_tutorial.

To ensure data quality, we applied a subset of tools developed by the Observational Health Data Science and Informatics (OHDSI) community to characterize the quality and content of OMOP-shaped datasets. In particular, we executed checks using the Achilles and DataQualityDashboard (DQD) packages to calculate high-level metrics about the ENCoDE data (e.g., age distributions) and its quality (e.g., plausibility and model conformance). We visualized the output from those packages across versions of the dataset using A Research Exploration System (ARES). This visual interrogation of the data enabled iterative feedback and informed subsequent updates of the dataset. For details, please see Table 3 and Fig. 4.

**Table 3:** Overview of data quality test performed.

	Verification Tests			Validation Tests			Total		
	Pass	Fail	Total	Pass	Fail	Total	Pass	Fail	Total
PlausibilityTests	2197	16	2213	287	0	287	2484	16	2500
Conformance Tests	919	7	926	145	0	145	1064	7	1071
Completeness Tests	446	2	448	17	0	17	463	2	465
Total	3562	25	3587	449	0	449	4011	25	4036

This table is an overview of all the tests performed by DQD and their categories on the ENCoDE datasets v1.0 on PhysioNet. Over 99% of tests are passed.

## Usage Notes

### Access

ENCoDE is available on PhysioNet as a credentialed database. To access the dataset, users must be registered on PhysioNet, have proper ethos training, and sign a data use agreement covering data usage and security standards, and prohibiting efforts to re-identify patients of the dataset.

### Limitations

The ENCoDE project was conducted in a single medical center, limiting the generalizability of our findings. Although our cohort included over 40% Black patients, the representation of the darkest skin tones was limited due to demographics in our community. Another limitation in this study is that we only include patients with pulse oximetry measurement five minutes or less before the ABG test. Also, different mobile cameras and mobile systems (iPhone or Android) could cause skin tone representations to be rendered differently.

## Data Availability

ENCoDE is available on PhysioNet as a credentialed database^20^. (https://physionet.org/content/encode-skin-color/1.0.0/).

## Code availability

Code for data extraction and preprocessing on PHI data is retained on internal Duke servers and is not available for redistribution. Code for processing the deidentified data is demonstrated on the corresponding PhysioNet page^20^ and on GitHub. We have created an official GitHub repository for the ENCoDE project. All the complementary tutorial codes and user support for technical problems will be stored here. https://github.com/aiwonglab/ENCoDE_tutorial.
